# Annotations

**Published:** 1895-02-23

**Authors:** 


### ANNOTATIONS.

**Wholeman Specialism.**

THE annual meeting of the British Medical Association, which is this year to be held in the metropolis, ought to be signalised by a large metropolitan philosophy and a capacity for dealing in a broad and statesman-like spirit with some of the most urgent problems of the hour in practical medicine. One of the questions which insistently demands consideration is the limitation of specialism in our art. Present day specialism —that is, the sole consideration of one organ or one part of the human body—can never play any but the most narrow *rôle* in a medicine which is really philosophical. If the human body were a mechanism only, in which each part was as absolutely complete in itself in nutrition and function as it is relatively complete in structure, we might then expect that some day every practitioner would be a specialist, and there would no longer be any *raison d'être* for the general physician. But nothing can be plainer in anatomy and physiology than that the parts exist for the whole, not the whole for the parts. Whatever, therefore, may be said in favour of a special study of separate parts may be said with tenfold more warrant in favour of a specialised study of the organism as a whole. In short, the time is not far distant, if medical culture is to advance, when the specialist of a part will no longer be known by the honoured title of "physician" or "surgeon." He will be a "dentist," or an "oculist," or a "cardiologist," or a "hepatist," or the like. All this is obvious to those medical men who reason philosophically. The matter is of much more importance than appears on the surface, and it is to be hoped that the members of the British Medical Association at their forthcoming meeting will take a large and competent view of the present situation, and so give an impulse to medicine in that rational direction which alone can make for true and general progress.

**Medical Journalism and Public Hospitals.**

IN a speech made at the last meeting of the Metropolitan Asylums Board, a point was raised which is of the greatest importance not only to their medical staff, but to all those whose lot it is to become patients within the walls of their hospitals. The point raised was as to the right of medical officers to communicate the results of their treatment to the medical press. Professor W. R. Smith is stated to have referred in terms of severe censure to the action of various superintendents in communicating "unauthorised" and "unofficial" statistics to the leading medical journals. The matter does not seem to have been taken up by any other speaker, but it is the thin end of a very large wedge, and looking at the question, as is our habit, from the points of view both of the public and of the profession, we must protest at once against any such attempt to muzzle the medical officers, or to prevent the freest expression of opinion, and even of professional criticism in the public press in regard to what goes on in what we feel justified in calling those dark places. The constitution of the Metropolitan Asylums Board is so peculiar; its power within its own sphere is so great; its facilities for raising money so large; and the information given to the public as to the expenditure of this money so very small, that we ought to hesitate greatly before we do anything to close up even one small rivulet by which the proceedings within the walls of their hospitals may trickle through to the public ear. We certainly do not wish any reports of treatment privately communicated to us, or to any of the other medical journals, to diminish by one jot the obligation which lies upon the Asylums Board to publish full and complete statistics as to its results, and statistics in such form that they can be usefully compared with those of the results in other hospitals. The compilation and comparison of the results from all the hospitals under the management of the Board is an affair which the central authority alone can undertake; but it is a matter of the extremest importance to the public, who entrust themselves and their children to these hospitals, to be assured that they are officered by men of science and humanity who will take an individual interest in their cases. This is best insured by giving the medical officers a free hand to do the best they can for their patients, to publish their results and to get the credit of them. The more individual the work, the greater the opportunities of publicity, and the more the medical officers of these hospitals are encouraged to lay their results freely before an appreciative but often critical profession, the greater will be the likelihood of the public obtaining good and careful treatment in these hospitals.

**The Pay System at the Great Northern Central.**

THE question of paying patients will be fought out at the annual meeting of the Governors of the Great Northern Central Hospital almost as we go to press. Dr. Glover is to move a resolution which is intended to sweep away the whole pay system root and branch. Mr. Burdett is to move an amendment, which will preserve the pay system; but preserve it for the advantage and convenience of those patients who may desire hospital treatment under the management of their own medical attendants. The situation is decidedly interesting. Some such pay system as that advocated by Mr. Burdett will probably have to be adopted, not only by the Great Northern Central Hospital, but by all the voluntary hospitals in the near future, if the hospitals are not to be thrust upon the rates. Dr. Glover is the advocate of a merely reactionary and *non possumus* policy: a policy which prevents patients who desire to be independent and thrifty from helping themselves and helping the hospitals. If Dr. Glover imagines that by leaving hospitals as they are he will either please the ratepayers or satisfy the general practitioners, he is very much mistaken. It is exactly the hospitals as they are that the general practitioner has his quarrel with. That quarrel is to some extent just. In many cases the nearest hospital is a competitor which the general practitioner finds quite intolerable. A movement of some kind must be made with the object of putting an end to this state of things. If Mr. Burdett's amendment is accepted, and a committee of the governors is appointed to deal with the pay question, we may confidently expect a solution which will satisfy both ratepayers and general practitioners. We propose to deal more fully with the question in our next issue, when the results of the governors' discussion will be available for comment.

